# Study protocol for the management of impacted maxillary central incisors: a multicentre randomised clinical trial: the iMAC Trial

**DOI:** 10.1186/s13063-022-06711-0

**Published:** 2022-09-16

**Authors:** Jadbinder Seehra, Andrew T. DiBiase, Shruti Patel, Rachel Stephens, Simon J. Littlewood, Richard J. Spencer, Tom Frawley, Philip E. Benson, Anthony J. Ireland, Farnaz Parvizi, Nikki Atack, Giles Kidner, Gabriella Wojewodka, Christopher Ward, Spyridon N. Papageorgiou, Jonathon T. Newton, Martyn T. Cobourne

**Affiliations:** 1 Centre for Craniofacial Development & Regeneration, Faculty of Dentistry, Oral & Craniofacial Sciences, King’s College London, Floor 27, Guy’s Hospital, London, SE1 9RT UK; 2 Maxillofacial Unit, William Harvey Hospital, Kennington Rd., Willesborough, Ashford, TN24 0LZ UK; 3 Department of Orthodontics, Kings College Hospital NHS Foundation Trust, Bessemer Road, London, SE5 9RS UK; 4 Orthodontic Department, St Luke’s Hospital, Little Horton Lane, Bradford, BD5 0NA UK; 5 Orthodontic Department, Pinderfields Hospital, Aberford Road, Wakefield, WF1 4DG UK; 6 Academic Unit of Oral Health and Development, School of Clinical Dentistry, Sheffield, S10 2TA UK; 7 Royal United Hospitals, Combe Park, Bath, Avon BA1 3NG UK; 8 Child Dental Health, Bristol Dental School, Lower Maudlin St, Bristol, BS1 2LY UK; 9 Orthodontic Department, Stoke Mandeville Hospital, Aylesbury, HP17 8UZ UK; 10 Research Portfolio Manager, King’s College London, Oral Clinical Research Unit, Faculty of Dentistry, Oral & Craniofacial Sciences (FoDOCS), Room 365, Floor 25 Tower Wing, Guys Hospital, Great Maze Pond, London, SE1 9RT UK; 11 South London CRN SSS Specialist and AcoRD Lead, NIHR Clinical Research Network (CRN), 16th Floor BRC Faculty, Guys Tower, Guys Hospital, London, SE1 9RT UK; 12 Clinic of Orthodontics and Pediatric Dentistry, Center of Dental Medicine, University of Zurich, Plattenstrasse 11, CH-8032 Zurich, Switzerland; 13 Population & Patient Health, Floor 18, Tower Wing, Guy’s Hospital, London, SE1 9RT UK

**Keywords:** Maxillary central incisor, Supernumerary tooth, Orthodontic space opening, Orthodontic traction, Randomised clinical trial, Unerupted, Eruption

## Abstract

**Background:**

Failure of eruption of the maxillary permanent incisor teeth usually presents in the mixed dentition between the ages of 7 and 9 years. Missing and unerupted maxillary incisors can be regarded as unattractive and have a potentially negative impact on facial and dental aesthetics. The presence of a supernumerary tooth (or odontoma) is commonly responsible for failed eruption or impaction of the permanent maxillary incisors. The primary objective of this trial is to investigate the success of eruption associated with maxillary incisor teeth that have failed to erupt because of a supernumerary tooth in the anterior maxilla.

**Methods:**

This protocol describes an interventional multicentre two-arm randomised clinical trial. Participants meeting the eligibility criteria will be randomised (unrestricted equal participant allocation [1:1]) to either space creation with an orthodontic appliance, removal of the supernumerary tooth and application of direct orthodontic traction or space creation with an orthodontic appliance, removal of the supernumerary tooth and monitoring. The primary outcome of this trial is to determine the prevalence of successfully erupted maxillary central permanent incisors at 6 months following removal of the supernumerary tooth. Secondary outcome measures include (1) the effect of initial tooth position (assessed radiographically) on time taken for the tooth to erupt, (2) time taken to align the unerupted tooth to the correct occlusal position, (3) gingival aesthetics and (4) changes in the self-reported Oral Health Related-Quality of Life (OHRQoL) (pre-and post-treatment).

**Discussion:**

There is a lack of high-quality robust prospective studies comparing the effectiveness of interventions to manage this condition. Furthermore, the UK national clinical guidelines have highlighted a lack of definitive treatment protocols for the management of children who present with an unerupted maxillary incisor due to the presence of a supernumerary tooth. The results of this trial will inform future treatment guidelines for the management of this condition in young children.

**Trial registration:**

ISRCTN Registry ISRCTN12709966. Registered on 16 June 2022.

**Supplementary Information:**

The online version contains supplementary material available at 10.1186/s13063-022-06711-0.

## Administrative information

Note: The numbers in curly brackets in this protocol refer to the SPIRIT checklist item numbers. The order of the items has been modified to group similar items (see above the administrative information table).

**Table:** 

Title {1}	Study protocol for the management of impacted maxillary central incisors: a multicentre randomised clinical trial: the iMAC Trial
Trial registration {2a and 2b}.	ISRCTN12709966
Protocol version {3}	Version 1 (1 November 2021)
Funding {4}	British Orthodontic Society Foundation. British Orthodontic Society, 12 Bridewell Place, London, EC4V 6AP
Author details {5a}	^1^Centre for Craniofacial Development & Regeneration, Faculty of Dentistry, Oral & Craniofacial Sciences, King’s College London, Floor 27, Guy’s Hospital, London, SE1 9RT, United Kingdom ^2^Maxillofacial Unit, William Harvey Hospital, Kennington Rd, Willesborough, Ashford TN24 0LZ, United Kingdom ^3^Department of Orthodontics, Kings College Hospital NHS Foundation Trust, Bessemer Road, London, SE5 9RS, United Kingdom ^4^Orthodontic Department, St Luke’s Hospital, Little Horton Lane, Bradford, BD5 0NA, United Kingdom ^5^Orthodontic Department, Pinderfields Hospital, Aberford Road, Wakefield, WF1 4DG, United Kingdom ^6^Academic Unit of Oral Health and Development, School of Clinical Dentistry, Sheffield S10 2TA, United Kingdom ^7^Royal United Hospitals, Combe Park, Bath, Avon, BA1 3NG ^8^Child Dental Health, Bristol Dental School, Lower Maudlin St, Bristol, BS1 2LY, United Kingdom ^9^Orthodontic Department, Stoke Mandeville Hospital, Aylesbury, HP17 8UZ, United Kingdom ^10^ Research Portfolio Manager, King’s College London, Oral Clinical Research Unit, Faculty of Dentistry, Oral & Craniofacial Sciences (FoDOCS), Room 365, Floor 25 Tower Wing, Guys Hospital, Great Maze Pond, London, SE1 9RT ^11^ South London CRN SSS Specialist and AcoRD Lead, NIHR Clinical Research Network (CRN), 16^th^ Floor BRC Faculty, Guys Tower, Guys Hospital, London SE1 9RT, United Kingdom ^12^Clinic of Orthodontics and Pediatric Dentistry, Center of Dental Medicine, University of Zurich, Plattenstrasse 11, Zurich, CH-8032 ^13^Population & Patient Health, Floor 18, Tower Wing, Guy’s Hospital, London SE1 9RT, United Kingdom
Name and contact information for the trial sponsor {5b}	Kings College London, 8^th^ Floor, Melbourne House, 44-46 Aldwych, London, WC2B 4LL (Professor Reza Razavi: reza.razavi@kcl.ac.uk) and Guys and St Thomas NHS Foundation Trust, Tower Wing, Great Maze Pond, London, SE1 9RT (R+D Email: R&D@gstt.nhs.uk)
Role of sponsor {5c}	This is an investigator-initiated and investigator-led trial. The funder of the trial has no role in trial design, data collection, data analysis or data interpretation.

## Introduction

### Background and rationale {6a}

Failure of eruption of the maxillary permanent incisor teeth usually presents in the mixed dentition between the ages of 7 and 9 years. Missing and unerupted maxillary incisors can be regarded as unattractive and have a potentially negative impact on facial and dental aesthetics, which may affect both self-esteem and social interaction [1]. The presence of a supernumerary tooth (or odontoma) is responsible for failed eruption or impaction of the permanent maxillary incisors in approximately 28–60% of cases [2–7]. In the UK, the prevalence of supernumerary teeth in the anterior maxilla has been reported at 2.6% and resulting in a failed eruption of 42% of central incisor teeth [8].

National clinical guidelines have highlighted a lack of definitive treatment protocols for the management of patients who present with an unerupted maxillary incisor due to the presence of a supernumerary [9]. There are no prospective randomised investigations comparing the effectiveness of both interventions. We propose to investigate whether there is any difference in the successful eruption and final alignment of unerupted maxillary central incisors associated with an unerupted supernumerary tooth following either removal of the supernumerary tooth, space creation and watchful waiting or removal of the supernumerary tooth, space creation and application of direct orthodontic traction in the first 6 months following surgery.

### Objectives {7}

The primary objective of this trial is to investigate the success of eruption associated with maxillary incisor teeth that have failed to erupt because of a supernumerary tooth (obstruction) in the anterior maxilla. Secondary objectives include the effect of initial tooth position (assessed radiographically) on time taken for the tooth to erupt, time taken to align the unerupted tooth to the correct occlusal position, gingival aesthetics and changes in the self-reported Oral Health Related-Quality of Life (pre- and post-treatment).

### Trial design {8}

This is a multicentre randomised clinical trial consisting of two parallel groups with equal randomisation to detect the superiority of one intervention over the other. Randomisation of the participants to one or two groups will be undertaken to ensure unrestricted equal participant allocation (1:1). This process will be undertaken centrally to ensure random allocation and concealment.

## Methods: participants, interventions and outcomes

### Study setting {9}

This protocol has been reported in adherence to the Standard Protocol Items: Recommendations for Interventional Trials (SPIRIT) guideline [10] (Additional file 1: Appendix 1). Potential participants will be recruited from those patients attending routine orthodontic treatment at the orthodontic department at each research collaborator/clinician hospital (secondary care) based in the UK.

### Eligibility criteria {10}

Participants meeting the following inclusion criteria will be included: aged between 8 and 10.5 (6 months to the day after their 10th birthday) years of age, fit and well, display optimal oral hygiene, presenting with the unilateral impaction of an upper maxillary central incisor due to the presence of a supernumerary tooth, be in the mixed dentition with the eruption of the upper first permanent molars, a single maxillary central incisor and lateral incisors and have parents who are able to give informed consent. Participants with either/or a history of previous orthodontic treatment, an impacted maxillary incisor due to root dilaceration or unfavourable root morphology; a simple space loss alone; participating in other trials or studies; a history of nickel allergy; and who decline to take part in the study will be excluded.

### Who will take informed consent? {26a}

Following the provision of both written information (Additional file 2: Appendix 2 and Additional file 3: Appendix 3) and verbal explanation to both the child and their parent, the dental care team (research team) member at each recruitment site will obtain consent from the parents of children who are suitable for inclusion in the study (Additional file 4: Appendix 4). Children will not be consented directly to the study as assent from children will also be obtained following liaison with their parents (Additional file 5: Appendix 5). If required, hospital-based interpreters based at each recruitment site will be made available through the NHS Trust on request. This is usually provided for patients who require an interpreter undergoing any routine orthodontic treatment. The participants taking part in this study are medically fit young children aged between 8.5 and 10.5 years of age who will be undergoing routine orthodontic treatment. In accordance with good clinical practice, assent with the child will be reconfirmed at each treatment visit. If the child’s parent lost capacity, then consent would be obtained from someone who has parental responsibility for the child.

### Additional consent provisions for collection and use of participant data and biological specimens {26b}

N/A. As part of the trial, no biological specimens will be collected.

### Interventions

#### Explanation for the choice of comparators {6b}

Early diagnosis and appropriate management are recommended for the unerupted maxillary incisor teeth. Following the removal of a supernumerary tooth, the retrospective evaluation suggests that between 49 and 91% of permanent maxillary incisors will erupt spontaneously [11–14]. Although these figures appear to be favourable, there is a large variation in the reported time taken for the incisor to erupt, which can be up to 18 months [12]. Eruption of the maxillary incisor can be facilitated by space creation in conjunction with the removal of the obstruction [7, 12, 14–18]. However, between 30 and 54% of the impacted incisors still require further surgical intervention [11, 13, 14, 19] and some form of orthodontic alignment [16]. In addition to the surgical removal of any obstruction, surgical exposure of the unerupted maxillary incisor may also be undertaken. In these circumstances, early orthodontic traction can enhance facilitated eruption [20]. Based on retrospective studies, the success of surgical exposure combined with orthodontic traction has been reported to exceed 90% [21]. However, the type of surgical exposure procedure undertaken in conjunction with orthodontic traction may affect both long-term gingival and periodontal outcomes of the erupted incisor [22].

#### Intervention description {11a}

Following informed consent, study participants will be allocated for routine orthodontic treatment with a pre-adjusted edgewise fixed appliance (0.022 × 0.028 in. slot size). A conventional upper sectional fixed appliance and mechanics will be utilised to open sufficient space within the dental arch to accommodate the unerupted maxillary central incisor. An upper sectional fixed appliance will be used to create the required space utilising a nickel titanium open coil spring placed on a 0.018-in. stainless steel archwire. Prior to surgery, the created space will be maintained using a passive closed coil spring placed in the space on a 0.018-in. stainless steel archwire. This should be equivalent to the mesio-distal width of the erupted contralateral maxillary central incisor. Following this, the participant will be randomised into two treatment groups using allocation concealment: surgical removal of the supernumerary tooth, gold chain bonding and immediate post-surgical orthodontic traction (group 1) or surgical removal of the supernumerary tooth only and monitoring eruption of the unerupted incisor for a period of 6 months (group 2).

In group 1, immediate application of piggyback orthodontic mechanics (0.014-in. nickel titanium or elastomeric traction and 0.018-in. stainless steel archwires) will be employed to erupt the tooth. Following the eruption of the incisal edge of the unerupted maxillary central incisor through the gingival mucosa, an attachment/orthodontic bracket will be placed onto the clinical crown to facilitate the final orthodontic alignment of this tooth. Piggyback mechanics (0.014-in. nickel titanium or elastomerics and 0.018-in. stainless steel archwires) will then be employed again to further erupt the tooth. In group 2, the eruption of the unerupted maxillary central incisor will be monitored and observed for 6 months. During this observation period, following the eruption of the incisal edge of the unerupted maxillary central incisor through the gingival mucosa, an attachment/orthodontic bracket will be placed to the clinical crown to facilitate the final orthodontic alignment of this tooth. Piggyback mechanics (0.014-in. nickel titanium or elastomerics and 0.018-in. stainless steel archwires) will then be employed again to further erupt the tooth. In both groups, the unerupted maxillary central incisor will be considered aligned once the correct occlusal level has been achieved compared to the contra-lateral maxillary central incisor and an upper 0.019 × 0.025-in. stainless steel archwire is ligated.

#### Criteria for discontinuing or modifying allocated interventions {11b}

In either group 1 or group 2, if after 6 months following removal of the supernumerary tooth the central incisor has failed to erupt, records (intra-oral photographs, study models and radiographs) will be taken. Following these records, a clinical decision will be made to either continue monitoring the eruption of the incisor, arrange a further surgical intervention or apply piggyback orthodontic mechanics (0.014-in. nickel titanium or elastomerics and 0.018-in. stainless steel archwires) to erupt the tooth.

#### Strategies to improve adherence to interventions {11c}

As with any routine orthodontic treatment, patients will be encouraged to attend on a regular basis for adjustment of the appliance and monitoring of treatment progress.

#### Relevant concomitant care permitted or prohibited during the trial {11d}

In both groups, any retained primary teeth in the upper arch will also be removed if indicated.

#### Provisions for post-trial care {30}

Following removal of the fixed appliance (T3) and 3-month post-treatment follow-up (T4), participants will be kept under review within the orthodontic department at each recruitment site as part of their routine care/follow-up. They will be assessed for the need for further routine orthodontic treatment based upon any underlying malocclusion.

### Outcomes {12}

The primary outcome of this investigation is to determine the prevalence of successfully erupted maxillary permanent central incisors at 6 months following removal of the supernumerary tooth (obstruction). As per previous prospective investigations of unerupted teeth, the primary endpoint of the study is defined as the eruption (successful outcome) of the unerupted maxillary central through the gingival mucosa during the 6-month observation period [23]. Clinically, the amount of clinical crown visible should *allow* the placement of an orthodontic attachment or removal of the bonded gold chain attachment and placement of an orthodontic attachment/bracket. Secondary outcome measures will include (1) the effect of initial tooth position (assessed radiographically) on the time taken for the tooth to erupt, (2) time taken to align the unerupted tooth to the correct occlusal position, (3) gingival aesthetics associated with the erupted central incisor and (4) changes in the self-reported Oral Health Related-Quality of Life (OHRQoL) (pre- and post-treatment).

### Participant timeline {13}

**Fig. 1:**
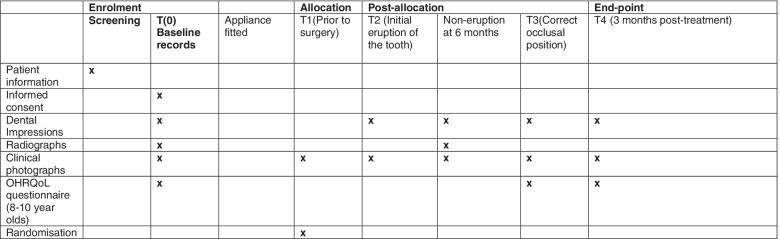
Schedule of enrolment, allocation, post-allocation and endpoint

The schedule of enrolment, allocation, post-allocation and endpoint is shown in Fig. 1 (participants will have further routine appointments for the adjustment of the fixed appliance at the following time points: appliance fitted, T2 non-eruption at 6 months and T3).

#### Sample size {14}

Sample size calculation was based on three previous studies [11, 12, 14], random-effects meta-analysis of which indicated that in 58% of cases (95% = 40–75%; *I*^2^ = 85%), the maxillary permanent central incisor erupts spontaneously after surgical removal of the supernumerary tooth. Assuming a moderate relative risk = 1.50 as clinically relevant, equal 1-1 distribution between the groups and aiming to find a difference using a chi-square test with *α* = 5% and *β* = 20% (power of 80%), a total of 40 patients per group would be needed. Adding an additional 15% for possible drop-outs, the aim was set at 46 patients/per group (92 in total).

#### Recruitment {15}

At each recruitment site, potential participants will be approached initially by a member of the dental care team (research team) in the diagnostic clinic, and if they express interest in participating, they will then be provided with the study information sheets (child and parental sheets) (Additional file 2: Appendix 2 and Additional file 3: Appendix 3). Patients will take the information sheet away and be invited to provide consent (Additional file 4: Appendix 4 and Additional file 5: Appendix 5) at their next dental appointment if they are willing to participate in the trial (records appointment (T0)). If required, hospital-based interpreters at each recruitment site will be made available through the NHS Trust on request. This is usually provided for patients who require an interpreter undergoing any routine orthodontic treatment. The study will not involve sensitive information. Subjects will be assigned a study number once they are recruited. Patients and parents declining to participate in the trial will be treated in the normal manner within each orthodontic department.

### Assignment of interventions: allocation

#### Sequence generation {16a}

Computer-generated randomisation will be undertaken centrally by the Kings Clinical Trials Unit (https://ctu.co.uk/randomisation/) which will assign participants to one of the two intervention groups to provide unrestricted equal participant allocation common for all (1:1).

#### Concealment mechanism {16b}

As the process will be undertaken centrally and independently from the clinical operators, this allows for allocation concealment of participants.

#### Implementation {16c}

The principal investigator at each site will contact the central randomisation site (Kings Clinical Trials) to determine the participant group allocation.

### Assignment of interventions: blinding

#### Who will be blinded {17a}

Treating clinicians and participants cannot be blinded to the treatment intervention; however, the statistician will be blinded to participant allocation. The assessor of records (radiographs) to determine the pre-treatment position (height and angulation) of the unerupted maxillary central incisor will also be blinded to the treatment allocation groups.

#### Procedure for unblinding if needed {17b}

Not applicable. Treating clinicians and participants cannot be blinded to the treatment intervention.

### Data collection and management

#### Plans for assessment and collection of outcomes {18a}

Data will be collected at five time points: (T0) pre-treatment (baseline) records: dental study casts, extra- and intra-oral photographs, radiographs, completion of the Quality of Life Questionnaire and patient and participant demographics; (T1) prior to randomisation to either surgical removal of the supernumerary tooth, gold chain bonding and immediate post-surgical orthodontic traction (group 1) or surgical removal of the supernumerary tooth and monitoring eruption of the unerupted incisor for a period of 6 months (group 2): intra-oral photographs; (T2) following eruption of the incisal edge of the unerupted maxillary central incisor through the gingival mucosa: dental study casts and extra- and intra-oral photographs; (T3) unerupted maxillary central incisor aligned to correct occlusal level compared to contra-lateral maxillary central incisor: dental study casts, extra- and intra-oral photographs and completion of the Oral Health-Related Quality of Life Questionnaire; and (T4) 3 months post-treatment: dental study casts, extra- and intra-oral photographs and completion of the Oral Health-Related Quality of Life Questionnaire.

The Oral Health-Related Quality of Life Questionnaire (8–10 years old) to be used in this trial is a previously validated instrument which has been used reliably in the UK population [24, 25]. In either intervention group, if after the observation period of 6 months following removal of the supernumerary tooth the central incisor has failed to erupt, the following records will be taken: intra-oral photographs, study models and radiographs.

#### Plans to promote participant retention and complete follow-up {18b}

All data will be collected by the dental care team at the recruitment sites and at participants’ routine orthodontic appointments. As with any routine orthodontic treatment, patients will be encouraged to attend on a regular basis for the adjustment of the appliance and monitoring of treatment progress.

#### Data management {19}

All personal data collected (data collection sheets and completed Quality of Life Questionnaires (OHRQoL)) will be stored under a controlled access in a clear filing system safe from flood, fire, burglary and pests in a locked cabinet at each recruitment site. One copy of the informed consent will be given to the patient and their parent, one copy will be kept in the medical notes and one copy to be placed retained by the research team which will be stored in a locked cabinet by a member of the research team’s office at each recruitment site. Participant consent forms will be kept separately from all other data collected. All research data will be stored in accordance with the Kings College London Records and Data Retention Schedule (3.40 primary medical research data should be stored for the completion of the project+ 10 years). The data will be stored in the chief investigators’ secure office (King’s College London Dental Institute). Information with regard to study subjects will be kept confidential and managed in accordance with the Data Protection Act, NHS Caldicott Guardian, the Research Governance Framework for Health and Social Care and Research Ethics Committee Approval. The minimum personal data will be retained after the end of the study. The recommended archiving length for paediatric research is 25 years for children. The data may be analysed later on for scientific validation of research, or for future research and audit. The Guy’s and St Thomas’ NHS Foundation Trust (GSTFT) and King’s College London (KCL) are co-sponsors of this research project and share Data Controller responsibilities. Where personal data is disclosed by GSTFT to KCL or vice versa, directly or indirectly to satisfy the requirements of the protocol, or for the purpose of monitoring or reporting adverse events, or in relation to a claim or proceeding brought by a participant in connection with the trial, KCL and GSTFT agree to comply with the obligations placed on a Controller by the Data Protection Legislation. This is not limited to, but includes, being responsible for and able to demonstrate compliance with the principles relating to the processing of personal data (Article 5 GDPR). GSTFT and KCL have outlined their data controller roles and responsibilities in an overarching Master Data Sharing Agreement.

#### Confidentiality {27}

All study participants will be pseudonymised and allocated a unique identification number known only to the PhD student and to the dental care team (research team) at each recruitment site. This number will be used to identify stone/plaster/digital models, extra- and intra-oral photographs taken of participant’s teeth and Quality of Life Questionnaires (OHRQoL). Pseudonym break Microsoft Excel spreadsheet will be held at GSTT/the local NHS sites on NHS password-protected computers. All clinical photographs will be stored on an NHS password-protected computer or uploaded onto each recruitment sites’ secure server. All email correspondence relating to the study participants between the recruitment sites will be sent using secure NHS email accounts. Personal data will be treated as confidential information under the guidance of the UK Data Protection Act, 2018 and the EU GDPR, 2018.

#### Plans for collection, laboratory evaluation and storage of biological specimens for genetic or molecular analysis in this trial/future use {33}

As part of the trial, no biological specimens will be collected.

## Statistical methods

### Statistical methods for primary and secondary outcomes {20a}

The primary outcome of the trial is the successful eruption (yes/no) of the impacted maxillary incisor, which is ascertained clinically in an objective matter and is clinically relevant (binary outcome). Secondary outcomes planned include (i) time taken for the maxillary incisor to erupt clinically after surgical removal of the supernumerary (continuous outcome in days), (ii) gingival aesthetics outcome according to Parkin et al. [26] (continuous outcome on a 100-cm visual analogue scale [VAS]) and (iii) patient-related outcomes included in the Oral Health-Related Quality of Life Questionnaire [24] (continuous scale outcome with total score and sub-scores for each category). Possible risk factors that are planned a priori to be investigated include patient age, patient sex, patient ethnicity, impacted incisor’s maturity, impacted incisor’s position (height, inclination and orientation; as described in the trial’s protocol) and the supernumeraries’ number or morphology (conical, premolariform, incisiform, complex odontoma, etc.). The initial crude differences between the groups will be assessed with the chi-square test for the primary outcome or the t-test for independent samples for the secondary outcomes (or non-parametric equivalents). The crude effect of intervention groups on the primary/secondary outcomes (with patient as the unit of analysis) will be further assessed with generalised linear models (after appropriate model diagnostics) with relative risk (RR) for the primary outcome or unstandardised regression coefficient for the secondary outcomes and their corresponding 95% confidence interval (CI). Furthermore, regression models will be adjusted for the potential impact of any of the above-listed confounders, by adding one covariate at a time in the simple model and retained if the change-in-estimate is at least 10% [27]. All analyses will be done per protocol by the statistician in a blind manner using a coded dataset in Stata 14.2 (StataCorp., College Station, TX, USA) with a significance level set at a two-sided P-value of 0.05 for all analyses.

### Interim analyses {21b}

Stopping of the trial could also be based on interim data analysis if, clearly, one treatment is better than the other, but no interim analysis is planned.

### Methods for additional analyses (e.g. subgroup analyses) {20b}

Except for the adjusted-for-confounders analyses detailed above, no other additional analyses or subgroup analyses are planned.

### Methods in analysis to handle protocol non-adherence and any statistical methods to handle missing data {20c}

Statistically significant RRs will be translated in a clinically relevant manner using risk differences and the number needed to treat. If multiple impacted teeth per patients are included, within-patient clustering will be taken into account with robust standard errors. If any centre effects are identified, these will be accounted for with a random term in the model.

### Plans to give access to the full protocol, participant-level data and statistical code {31c}

The protocol will be published on a publicly accessible database, ISRCTN Registry (https://www.isrctn.com/). The full anonymised dataset of the trial will be made openly available through Zenodo.

### Oversight and monitoring

#### Composition of the coordinating centre and trial steering committee {5d}

A monitoring/steering/safety committee will not be set up for this study. The team at the Kings Clinical Trials Unit will be responsible for the randomisation of participants to the trial arms. Each primary investigator and their dental care team at each recruitment site will be responsible for local organisation of the trial including identifying potential recruits and taking informed consent and data collection. The trial will be supervised by the chief investigator who will organise meetings every 4–6 months with the primary investigators at each recruitment site to discuss the progress of the trial and any operational or clinical challenges that have be encountered. A stakeholder and public involvement group (SPIG) was used to support the justification of the trial during the ethical approval process. However, a SPIG will not be required during the conduct of the trial.

#### Composition of the data monitoring committee, its role and reporting structure {21a}

A monitoring/steering/safety committee will not be set up for this study. The chief investigator will be responsible for the ongoing management of the study. A data monitoring committee is not required as this is a low-risk intervention (comparison of existing standards of care), and the ethics committee did not request a data monitoring committee.

#### Adverse event reporting and harms {22}

No serious adverse events (SAEs) are expected to occur as part of this trial. Participants enrolled in the trial will be undergoing routine orthodontic treatment within the orthodontic departments of each recruitment site, and this treatment does not differ from any other patients that are treated in the respective departments. Subject safety will be assessed for all patients undergoing treatment within the department (physical examination, adverse event reporting). Where an SAE is related to the study procedures or is an unexpected occurrence, then it will be reported immediately upon knowledge of the event to Guy’s and St Thomas’ R&D within 24 h. For all other AEs, these will be reported to R&D when copied into the Annual Progress Report. The principal investigators at all sites must report all SAEs to the chief investigator first where possible. The chief investigator is then responsible for reporting events to R&D.

#### Frequency and plans for auditing trial conduct {23}

The sponsor will monitor and conduct audits on a selection of studies in its clinical research portfolio. Monitoring and auditing will be conducted in accordance with the UK Policy Framework for Health and Social Care 2017 and in accordance with the sponsor’s monitoring and audit procedures. This study may be identified for audit by any of the following indications: project may be identified via the risk assessment process, an individual investigator or department may request an audit, a project may be identified via an allegation of research misconduct or fraud or a suspected breach of regulations, projects may be selected at random as per the Department of Health which recommends that trusts should be auditing a minimum of 10% of all research projects and projects may be randomly selected for audit by an external organisation and internal audits conducted by a sponsor’s representative.

#### Plans for communicating important protocol amendments to relevant parties (e.g. trial participants, ethical committees) {25}

If required, any protocol amendments will be communicated to local R+D departments and subsequent ethics committees. Following this, a revised protocol would be sent to the PI at all recruitment sites. The clinical trial register will also be updated.

#### Dissemination plans {31a}

The chief investigator, PhD student or treating dental care team (research team) at each recruitment site will inform the patients about the study findings after the trial ends. A summary sheet regarding the results of the study will be provided. The reporting of this trial will be in accordance with the CONSORT guidelines [28]. The results of the study will be disseminated via appropriate scientific publications (peer-reviewed journals) and presentations at conferences/meetings. Additionally, a printed copy of the accepted scientific publication following completion of the study will be posted to each participant (optional). The use of professional writers will not be employed.

## Discussion

This trial was planned to commence prior to 2020. However, given the disruption caused to clinical services by the COVID-19 pandemic, a delay in commencing would appear advantageous, but challenges still remain. The recruitment of participants into this trial is currently limited to secondary care services (orthodontics, paediatrics and oral survey) which are under pressure to reduce patient treatment waiting lists and restoring clinical activity to pre-COVID levels. The participants with the index condition for this trial are deemed a high need for treatment as per the Index of Orthodontic Treatment Need (IOTN) [27] and are commonly referred from primary care to second care environments as a multi-disciplinary management is often required. On this basis, we do not anticipate difficulty in achieving the required sample size as recruitment of patients is being undertaken across nine sites. However, future rises in COVID-19 infections could impact recruitment especially if national restrictions are reactivated and staff at recruitment sites are redeployed. To manage potential recruitment issues, the inclusion of additional recruitment sites will be considered.

As encountered by other clinical trials in orthodontics, blinding of both participants and investigators at recruitment sites will not be possible. To minimise bias, the randomisation (allocation and concealment) will be undertaken centrally at a Clinical Trials Unit. Furthermore, both the investigator assessing the clinical records and the statistician will be unaware of the participant trial arm allocation.

### Trial status

Protocol version 1 (01/11/2021). Recruitment is anticipated to begin on 1 July 2022 and end 6 months before the end of the trial (01/11/2024).

## Supplementary Information

**Additional file 1: Appendix 1.** SPIRIT Checklist for Trials.

**Additional file 2: Appendix 2.** Parent /Guardian Patient information Sheet – The iMAC Trial.

**Additional file 3: Appendix 3.** Child/Young person Patient information Sheet – The iMAC Trial.

**Additional file 4: Appendix 4.** Parent /Guardian Consent form– The iMAC Trial.

**Additional file 5: Appendix 5.** Child/Young person Assent Form– The iMAC Trial.

## Data Availability

Members of the direct care team will have access to the personal data. This also includes a PhD student who is part of the direct care team. Regulatory authorities and NHS R&D offices at each recruitment site will monitor and conduct audits on a selection of studies in its clinical research portfolio.
