# Supplementary material for: Study protocol for the management of impacted maxillary central incisors: a multicentre randomised clinical trial: the iMAC Trial
Source: Trials. 2022 Sep 16;23:787. doi: 10.1186/s13063-022-06711-0 (PMC9479226; doi:10.1186/s13063-022-06711-0)
Supplement: Supplementary file 2 — Additional file 2: Appendix 2. Parent /Guardian Patient information Sheet – The iMAC Trial. [file 13063_2022_6711_MOESM2_ESM.docx]

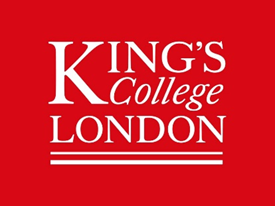


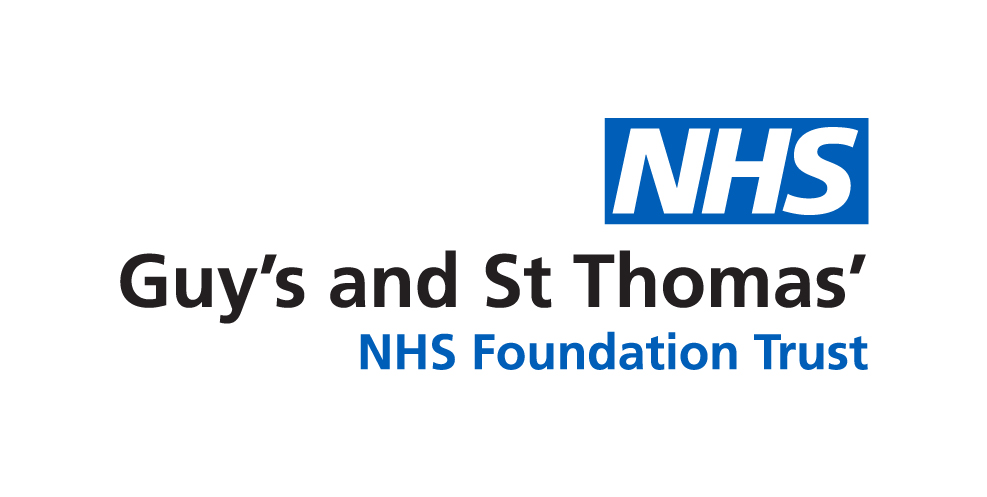


**King’s College London Dental Institute**

**Guy’s and St Thomas’ NHS Foundation Trust**

**Parent/Guardian Information Sheet**

Version 2 (10/03/2022)

The iMAC Trial **(**Management of **i**mpacted **MA**xillary **C**entral incisors)

Name of Researcher: Professor Martyn Cobourne

**Invitation**

We are inviting you and your child to take part in a research study. Before you decide it is essential that you understand why the research is being done and what it will involve. One of our team will go through this information sheet with you in detail and answer any questions that you might have. Please take the time to read the following information carefully. Talk to others about the study if you wish. Please ask us anything that is not clear or if you would like more information. Part 1 tells you the purpose of this study and what will happen if they take part. Part 2 gives you more detailed information about the conduct of the study.

**Part 1**

**What is the purpose of this study?**

Failure of a front tooth in the top jaw to erupt is a common problem in young children affecting approx. 3% of the population. The consequence of this disturbance in dental development can cause the persistence of spacing between the teeth, an unbalanced and unattractive smile appearance and compromised dental aesthetics. More importantly, the impact of not having a front tooth in the top jaw present can cause a negative impact on a child’s self-esteem, social interaction and potentially predispose a child to bullying episodes.

In the United Kingdom, in about 50% of cases the cause for the failure of the front tooth to erupt into its normal position is the presence of a supernumerary or extra tooth. This extra tooth is a physical obstruction, which impedes the normal eruption of the top front tooth. In this clinical situation, it is very unlikely that the top front tooth will erupt spontaneously and hence treatment is often required.

Two standard treatment approaches are considered in this clinical situation. The first is to remove the extra tooth under General anaesthetic and monitor the eruption of the unerupted top front tooth. In scientific studies, the success of this treatment has been reported to range between 49-91%. The second treatment option is to use orthodontic braces in conjunction with surgical removal of the extra tooth, uncovering and bonding of a gold chain attachment to the unerupted top front tooth to align it into its correct position. The success of this treatment approach has been reported to around 90%. Although these figures appear to be favourable, there is large variation in the reported time taken for the top front tooth to erupt, which can be up to 18 months.

The disadvantages of the first approach are that the time taken for the tooth to spontaneously erupt is very variable and it may erupt into a malaligned position, hence requiring further treatment. The second approach is associated with more predictability in terms of the time taken to align the tooth but may result in a possible unesthetic alteration of the natural gumline around the tooth.

The scientific evidence base behind both treatments is unclear and we do not know which approach is more successful in terms of successfully erupting the tooth and which approach results in better gum line aesthetics and ultimately an aesthetically pleasing smile.

This study aims to compare two treatment types (removal of the extra tooth only or removal of the extra tooth and immediate application of orthodontic brace forces) in facilitating successful eruption of the top front tooth. If you allow your child to participate in this study, he/she will be randomised to one of these two treatments. The results of this study will contribute to the establishment of treatment guidelines based on high quality evidence, development of efficient treatment protocols and high-quality outcomes.

This study will be led by the Chief investigator, Professor Martyn Cobourne, Hon Consultant in Orthodontics, King’s College London Dental Institute and Guy’s and St Thomas’ NHS Foundation Trust. The project also forms part of a King’s College London PhD project in clinical dentistry.

**Why have I been invited?**

Your child has been invited to participate because they have an impacted incisor (unerupted top front tooth) and will be undergoing orthodontic treatment within this department.

**Do they have to take part?**

No, it is up to you and your child to decide whether or not they take part. If you decide to participate, you will both be given an information sheet to keep with you and will be asked to sign a consent form at your child next appointment. Your child will also be asked to sign an assent form, indicating that they are happy to take part. You are still free to withdraw from the study at any time without giving a reason. A decision to withdraw at any time, or a decision not to take part will not affect the standard of care you receive. If you or your child decide to withdraw from the study, data already collected as part of the study will be retained, but no further information will be taken.

**What will happen if we decide to take part?**

If you and your child agree to take part, the researchers will obtain informed consent initially. The first stage would involve taking routine orthodontic records consisting of dental impressions of the teeth, photographs of your child’s face and teeth and appropriate radiographs (x-rays) which are used to identify the extra tooth (supernumerary tooth) and locate the position of the unerupted maxillary incisor (top front tooth). These records will be updated during your child’s treatment at certain points. In addition, your child will be asked to complete a short questionnaire at various points during the study, which assesses how the unerupted maxillary incisor (top front tooth) and treatment affects their overall well-being (Quality of life). This short questionnaire will take your child 10 minutes to complete.

All children who take part in this study, will have an upper orthodontic appliance (top fixed metal brace) placed initially to first open space between the teeth for the unerupted maxillary incisor (top front tooth). Your child will be seen at 4-6 week intervals for the routine adjustment of their upper orthodontic appliance (fixed brace).

Once sufficient space has been created, your child will be randomly allocated into one of two treatment groups using computer software (Graphpad). In the first group, your child will be referred to our oral surgery team for the surgical removal of the extra tooth (supernumerary tooth) and the removal of any primary (baby) teeth only (Standard care). The eruption of the unerupted maxillary incisor (top front tooth) is then monitored over for a period of 6 months allowing natural eruption of the tooth. Your child will be seen for the routine adjustment of their upper orthodontic appliance (fixed brace) every 4-6 weeks. If after 6 months, the unerupted maxillary incisor (top front tooth) has failed to erupt we will update the clinical records and a decision will be made to either continue monitoring the eruption of the tooth or arrange a small surgical procedure to uncover the tooth and place a gold chain attachment. Using the orthodontic appliance (top fixed brace) we will start to apply gentle forces via the gold chain attachment on the unerupted maxillary incisor (top front tooth). During this period, once the tooth is visible through the gingival tissues (gum) we will place an attachment on it and using the orthodontic appliance (top fixed brace), guide it into the correct position in the front of your child’s mouth.

In the second group (Intervention being assessed), your child will be referred to our oral surgery team for the surgical removal of the extra tooth (supernumerary tooth) and the removal of any primary (baby) teeth only and the surgical uncovering and placement of attachment (gold chain) on the unerupted maxillary incisor (top front tooth). Using the orthodontic appliance (top fixed brace) we will immediately start to apply gentle forces via the gold chain attachment on the unerupted maxillary incisor (top front tooth). Your child will be seen for the routine adjustment of their upper orthodontic appliance (fixed brace) every 4-6 weeks. During this period, once the tooth is visible through the gingival tissues (gum) we will place an attachment on it and using the orthodontic appliance (top fixed brace), guide it into the correct position in the front of your child’s mouth.

All surgical procedures are commonly undertaken as a Daycase procedure under General Anesthesia. The orthodontic appliance (top fixed brace) will remain in place during this procedure. Both groups will receive the same orthodontic treatment with the same top fixed brace. The only difference is in one group will have a gold chain placed on the unerupted maxillary incisor (top front tooth) following surgical removal of the extra tooth (supernumerary tooth). While in the other group we will monitor the natural eruption of the unerupted maxillary incisor (top front tooth) without a gold chain attachment for a period of 6 months. Both groups will receive standard treatment, which is no different from any other patient in our department.

In either group, there is a small risk for the need for further surgical interventions to either place a gold chain onto the unerupted tooth if it fails to erupt after 6 months or indeed reattaching the gold chain to tooth as it can sometimes become loose when we aligning the tooth with braces. This may involve another General anesthetic but based on your child’s compliance, any potential further surgical intervention in both groups could be performed under local anaesthesia with or without sedation.

**Will my child or I receive compensation for participation in this study?**

No payments or travel expenses will be paid if you participate.

**How will we use information about you?**

We will need to use information from your child’s medical records for this research project.

This information will include your child’s initials, NHS number, name, date of birth contact details and hospital number.

The orthodontic clinician, clinical staff and researchers will use this information to do the research or to check your child’s records to make sure that the research is being done properly.

Clinicians and staff such as a statistician who do not need to know who your child is, will not be able to see your child’s name or contact details. Your child’s data will have a code number instead. We will keep all information about your child safe and secure.

Once we have finished the study, we will keep some of the data so we can check the results. We will write our reports in a way that no-one can work out that your child took part in the study.

**What are your choices about how your information is used?**

Your child can stop being part of the study at any time, without giving a reason, but we will keep information about your child that we already have.

If you choose to stop taking part in the study, we would like to continue collecting information about your child’s treatment from child’s NHS records. If you do not want this to happen, tell us and we will stop.

We need to manage your records in specific ways for the research to be reliable. This means that we won’t be able to let you see or change the data we hold about you.

**Where can you find out more about how your information is used?**

You can find out more about how we use your information

- at [www.hra.nhs.uk/information-about-patients/](https://www.hra.nhs.uk/information-about-patients/)
- our leaflet available from: [www.guysandstthomas.nhs.uk/research/patients/use-of-data.aspx](http://www.guysandstthomas.nhs.uk/research/patients/use-of-data.aspx) (For GSTT) and [www.kcl.ac.uk/research/support/research-ethics/kings-college-london-statement-on-use-of-personal-data-in-research](http://www.kcl.ac.uk/research/support/research-ethics/kings-college-london-statement-on-use-of-personal-data-in-research) (for KCL)
- by asking one of the research team (contact details included below)
- by contacting the Data Protection Officer: (For GSTT: Nick Murphy-O’Kane [DPO@gstt.nhs.uk](mailto:DPO@gstt.nhs.uk); For KCL: Albert Chan [info-compliance@kcl.ac.uk](mailto:info-compliance@kcl.ac.uk))

**Will you inform my child’s General Dental Practitioner (GDP) of their participation in the study?**

With your permission, a letter will be sent to your dentist (General Dental Practitioner) to let them know about the participation of your child in the study.

**What are the other possible disadvantages and risks of taking part?**

There are no additional disadvantages or risks of taking part in this study. Your child’s orthodontic treatment will be the same for any patient who presents with impacted teeth. Any routine orthodontic brace treatment can be associated with some pain and discomfort around the teeth, particularly for a few days after the wires are changed. This is managed with normal appliance care instructions, which are provided when your child has his/her brace fitted. There will be some minimal inconvenience, related to completing questionnaires but completion of these questionnaires should not take more than 10 minutes. Also additional chair- side time is required to take impressions of the upper teeth and clinical intra-oral photographs. In some cases, we may need to arrange additional x-rays which results in further exposure to ionising radiation. To reduce these inconveniences, the impression and photographs are to be taken by fully trained clinicians.

Thank you for reading so far – if you are still interested, please go to Part 2:

**Part 2**

**What happens if new information about the research comes along?**

Sometimes during research, new scientific findings are reported in the literature. Your clinician/orthodontist will inform you and your child about this if it happens.

**What will happen to the records collected during the study?**

During the research study all information collected about your child will be stored in secure locations and on password protected computers. After the study is finished, the models, photographs, questionnaires and data will be kept for 25 years after the study has ended in accordance with the Trust policy for managing children’s confidential data. All patient identifiable data will be stored at Kings College London and deleted at the end of the study. This data will be achieved at Guy’s and St Thomas’ NHS Foundation Trust.

**Who has reviewed this study?**

All research in the NHS is looked at by independent group of people, called a Research Ethics Committee to protect you and your child’s safety, rights, wellbeing and dignity. They also make sure that the research is fair and appropriate. A Patient and Public (PPI) group has also reviewed the study patient information sheets.

**What happens if there is a problem?**

If you have a concern about any aspect of this study, you should ask to speak to the researchers who will do their best to answer your questions. Please contact the Principal Investigator: XXXXXXXXXX; Tel: XXXXXXXXXX; email: XXXXXXXXXXX. If you remain unhappy and wish to complain formally, you can also do this through the Patients Advice and Liaison Service (PALS) at the hospital where your child is having their orthodontic treatment.

In the event that something does go wrong, and you are harmed during the research you may have grounds for legal action for compensation against Guy’s and St Thomas’ NHS Foundation Trust and/or King’s College London, but you may have to pay your legal costs. The normal National Health Service complaints mechanisms will still be available to you (if appropriate).

With your permission, a printed copy of the results of the study (scientific publication) will be posted to you following completion of the study.

**Thank you for reading this. Please ask any questions if you need to.**

Professor Martyn Cobourne (Chief investigator)

Department of Orthodontics, Floor 27, King’s College London Dental Institute, Guy’s Tower, Guys and St Thomas NHS Foundation Trust, Great Maze Pond, London SE1 9RT

Telephone: 02071884415

Email: Martyn.Cobourne@kcl.ac.uk

Dr Jadbinder Seehra (Investigator and PhD student)

Department of Orthodontics, Floor 25, King’s College London Dental Institute, Guy’s Tower, Guys and St Thomas NHS Foundation Trust, Great Maze Pond, London SE1 9RT

Telephone: 02071884415

Email: Jadbinderpal.Seehra@kcl.ac.uk

If you would prefer to ask questions to someone who is familiar with and able to discuss the study, but not part of the study team. Please contact:

Miss Janine Smith

Programme Officer, Faculty of Dentistry, Oral & Craniofacial Sciences, King’s College London

Administrative Office, Postgraduate Centre, Floor 22, Guy's Hospital

London SE1 9RT
Telephone: 0207 1887188 Ext: 84415

Email: janine.smith@kcl.ac.uk

If you have any concerns or worries about your child’s treatment, then you can also contact:

Patient Advice and Liaison Service (PALS)
St Thomas' Hospital
Westminster Bridge Road
London SE1 7EH

Telephone: 020 7188 8801

Email: pals@gstt.nhs.uk
