# Supplementary material for: Study protocol for the management of impacted maxillary central incisors: a multicentre randomised clinical trial: the iMAC Trial
Source: Trials. 2022 Sep 16;23:787. doi: 10.1186/s13063-022-06711-0 (PMC9479226; doi:10.1186/s13063-022-06711-0)
Supplement: Supplementary file 3 — Additional file 3: Appendix 3. Child/Young person Patient information Sheet – The iMAC Trial. [file 13063_2022_6711_MOESM3_ESM.docx]

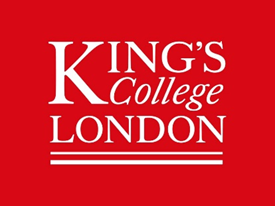
 **
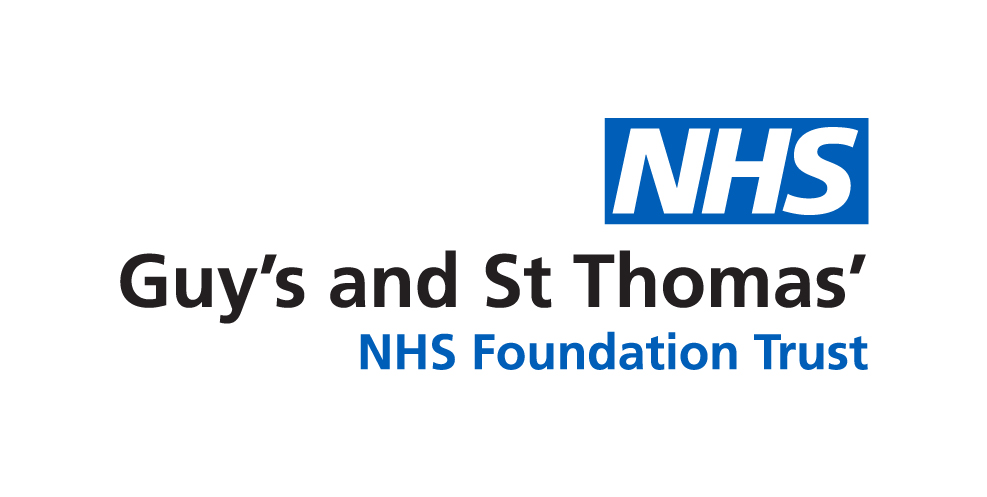
**

**King’s College London Dental Institute**

**Guy’s and St Thomas’ NHS Foundation Trust**

**Child/Young Person Information Sheet (8-10.5 years old)**

Version 2 (10/03/2022)

The iMAC Trial (Management of **i**mpacted **MA**xillary **C**entral incisors)

Name of Researcher: Professor Martyn Cobourne

**Invitation**

You are being invited to take part in a research project. Before you decide, it is important you know why the research is being done and what it would involve from you. Please read the following information carefully and discuss it with others if you wish. Ask us if there is anything that is not clear or if you would like more information.

**Why are we doing this research?**


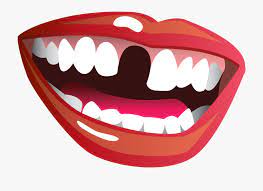


Some children like you may have a space between your top front teeth because your top front tooth is stuck in the gum.

An extra tooth blocking its path commonly causes this. We want to find out which treatment is better to try and get your top front tooth in the correct position so you do not have a space between your top front teeth.

**Why me?**

You have been invited to take part because you have an upper incisor tooth (top front tooth), which is stuck, in the gum due to an extra tooth (supernumerary tooth) blocking its path.

**Do I have to take part?**

No, it is completely up to you. If you do choose to take part, we will ask you to sign a permission form (assent form) to let us know you are happy to take part in the research project. We will give you a copy of this information sheet and your signed forms to keep. If you change your mind, you are free to pull out at any time, without giving a reason. If you decide you don’t want to take part, that’s fine. You will still receive the care you would have normally.

**What will happen to me if I do take part?**


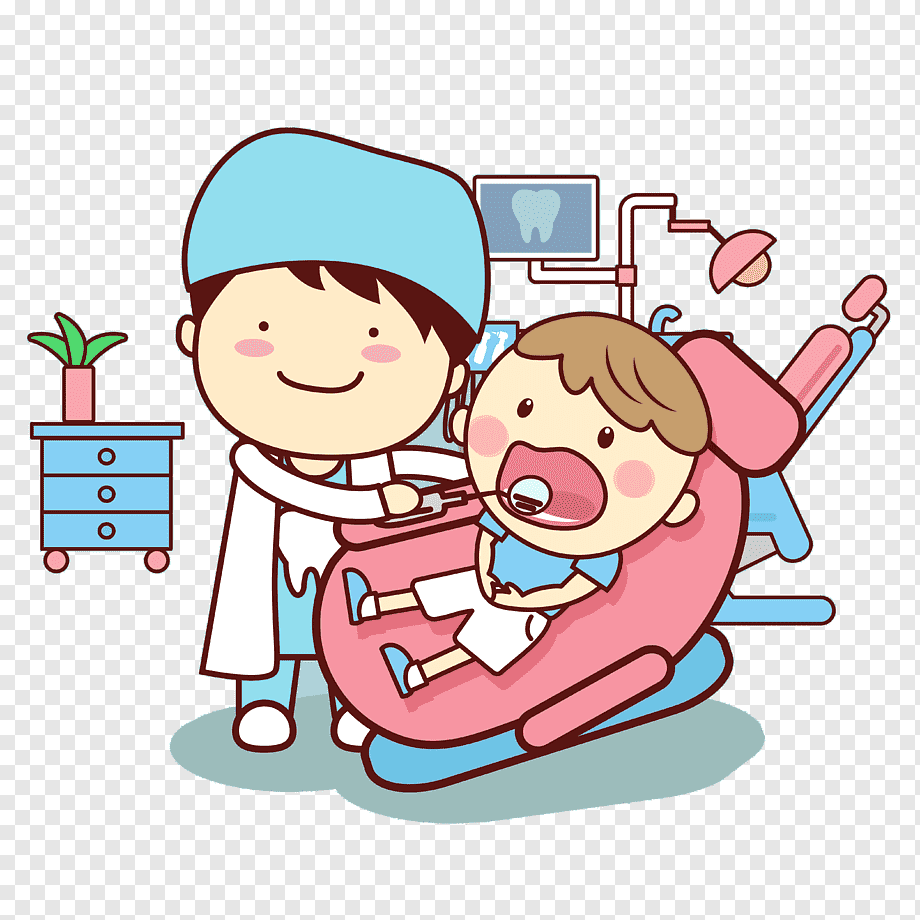


If you and your parents agree to take part, you will be involved in this study.

At certain points during the treatment we will take impression moulds of your teeth, photographs of your face and teeth and x-rays to check the position of the top front tooth under the gum and the extra tooth.


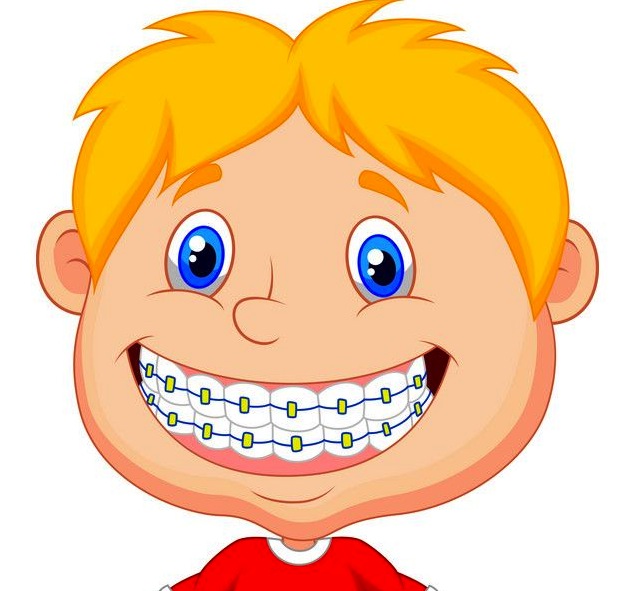


You will then be placed into one of two groups. The researchers will randomly decide which group you are part of.

After this you will have an operation to take out the extra tooth only or maybe a small gold chain will also be placed on the tooth stuck in the gum.

In one group we will just watch and monitor you for 6 months to see if the top tooth stuck in the gum comes down by itself.

If after this time, the tooth does not come down; we will either continue to monitor the tooth or arrange for small gold chain placed on the tooth. This may involve another operation. This chain is used to guide the tooth into the correct position in the front of your mouth.

In the other group, we use the top metal brace (with the gold chain) straightway after your operation to gently pull on the tooth with the chain on it to guide it into the correct position in the front of your mouth.

Normally as part of this treatment you will have a fixed orthodontic metal brace placed on your top teeth. This is used to make space for the tooth, which is stuck.

**What will I be asked to do?**

When you are having this treatment you will be seen every 4-6 weeks by your orthodontist to check your brace but also to see if the top front tooth under the gum is moving.

Any child having a brace is also asked to keep their teeth and brace very clean and to avoid breaking their brace by eating hard or chewy foods.

We will also ask you to fill out a questionnaire on a few occasions, which asks you how you feel about the top tooth stuck in the gum and your treatment.


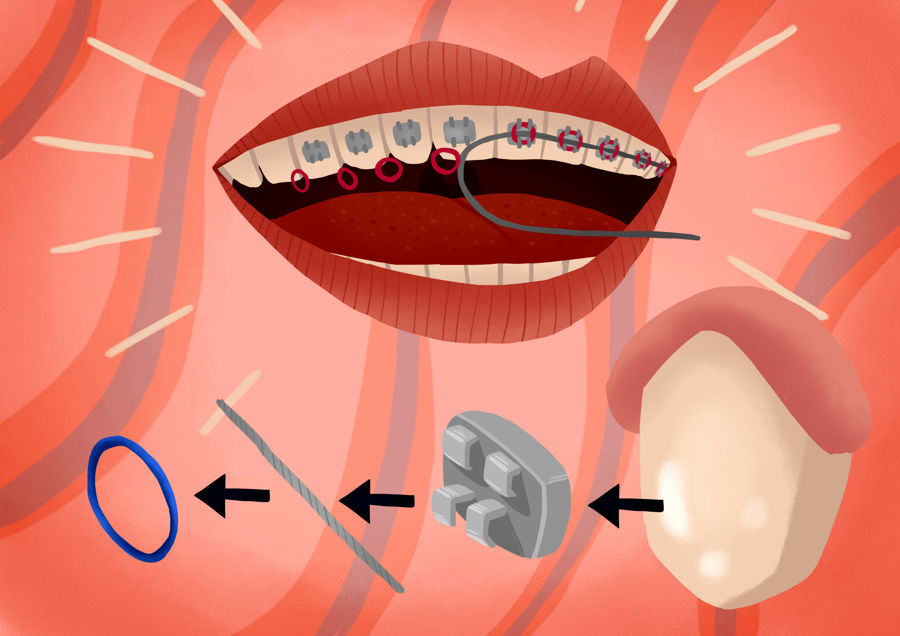


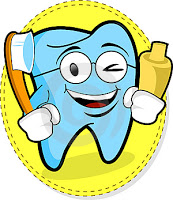


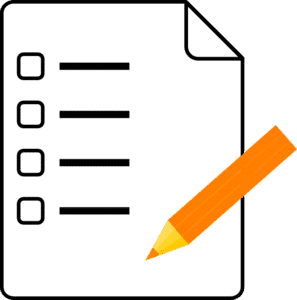


**Will my taking part in this study be kept confidential?**

Yes. All information that is collected about you during the course of the research will remain private and will be available to only those who need to see it. Your own dentist (GDP) will also know your taking part in this project. The information held about you will include the results of the questionnaires, brace treatment progress, your age and gender (boy or girl). This information will be kept in such a way that it is not linked to your name or any information that can identify you.

**Is there anything else to be worried about if I take part?**

There is no reason to be worried. Your orthodontic treatment will be the same for any child who presents with a tooth which is stuck in the gum. Any routine orthodontic brace treatment can cause some pain and discomfort around the teeth, particularly for a few days after the wires are changed. This is managed with normal appliance care instructions, which are given to you when your brace is fitted. During your treatment, on a few occasions we will ask you fill out questionnaires, take impressions moulds of your teeth, x-rays and photographs of your teeth. These may take up 10 minutes to do and will be performed by your orthodontist.

**What are the possible benefits of taking part?**

Although taking part in this research will not change your treatment, we hope that by taking part you will be helping us to understand better how to treat young children like you who have an upper incisor tooth (top front tooth) which is stuck, in the gum.

**What if there is a problem or something goes wrong?**

You will always be able to contact the department or the orthodontist who is doing your treatment to discuss your concerns and/or to get help.

**What are your choices about how your information is used?**

Your can stop being part of the study at any time, without giving a reason, but we will keep your information about you that we already have.

If you choose to stop taking part in the study, we would like to continue collecting information about your treatment from your NHS records. If you do not want this to happen, tell us and we will stop.

We need to manage your records in specific ways for the research to be reliable. This means that we won’t be able to let you see or change the data we hold about you.

**Where can you find out more about how your information is used?**

You can find out more about how we use your information

- at [www.hra.nhs.uk/information-about-patients/](https://www.hra.nhs.uk/information-about-patients/)
- our leaflet available from: [www.guysandstthomas.nhs.uk/research/patients/use-of-data.aspx](http://www.guysandstthomas.nhs.uk/research/patients/use-of-data.aspx) (For GSTT) and [www.kcl.ac.uk/research/support/research-ethics/kings-college-london-statement-on-use-of-personal-data-in-research](http://www.kcl.ac.uk/research/support/research-ethics/kings-college-london-statement-on-use-of-personal-data-in-research) (for KCL)
- by asking one of the research team (contact details included below)
- by contacting the Data Protection Officer: (For GSTT: Nick Murphy-O’Kane [DPO@gstt.nhs.uk](mailto:DPO@gstt.nhs.uk); For KCL: Albert Chan [info-compliance@kcl.ac.uk](mailto:info-compliance@kcl.ac.uk))

**What will happen to the records and information I give?**

During the research project all information collected about you will be stored in secure locations and on password protected computers. Usually, because you are young we keep this information for 25 years once the project has ended. After the study is finished, the models of your teeth, photographs, questionnaires and data will all be destroyed. All patient identifiable data will be stored at Kings College London and deleted at the end of the study. This data will be achieved at Guy’s and St Thomas’ NHS Foundation Trust.

If you agree, a printed copy of the results of the study (scientific publication) will be posted to you following completion of the study.

**Thank you for reading this. Please ask any questions if you need to.**

Professor Martyn Cobourne (Chief investigator)

Department of Orthodontics, Floor 27, King’s College London Dental Institute, Guy’s Tower, Guys and St Thomas NHS Foundation Trust, Great Maze Pond, London SE1 9RT

Telephone: 02071884415

Email: Martyn.Cobourne@kcl.ac.uk

Dr Jadbinder Seehra (Investigator and PhD student)

Department of Orthodontics, Floor 25, King’s College London Dental Institute, Guy’s Tower, Guys and St Thomas NHS Foundation Trust, Great Maze Pond, London SE1 9RT

Telephone: 02071884415

Email: Jadbinderpal.Seehra@kcl.ac.uk

If you would prefer to ask questions to someone who is familiar with and able to discuss the study, but not part of the study team. Please contact:

Miss Janine Smith

Programme Officer, Faculty of Dentistry, Oral & Craniofacial Sciences, King’s College London

Administrative Office, Postgraduate Centre, Floor 22, Guy's Hospital

London SE1 9RT
Telephone: 0207 1887188 Ext: 84415

Email: [janine.smith@kcl.ac.uk](mailto:janine.smith@kcl.ac.uk)

If you have any concerns or worries about your treatment, then you can also contact:

Patient Advice and Liaison Service (PALS)
St Thomas' Hospital
Westminster Bridge Road
London SE1 7EH

Telephone: 020 7188 8801

Email: pals@gstt.nhs.uk
