# Supplementary material for: Study protocol for the management of impacted maxillary central incisors: a multicentre randomised clinical trial: the iMAC Trial
Source: Trials. 2022 Sep 16;23:787. doi: 10.1186/s13063-022-06711-0 (PMC9479226; doi:10.1186/s13063-022-06711-0)
Supplement: Supplementary file 4 — Additional file 4: Appendix 4. Parent /Guardian Consent form– The iMAC Trial. [file 13063_2022_6711_MOESM4_ESM.docx]

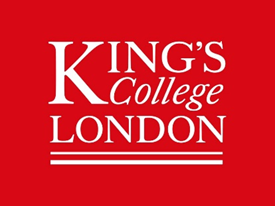


**
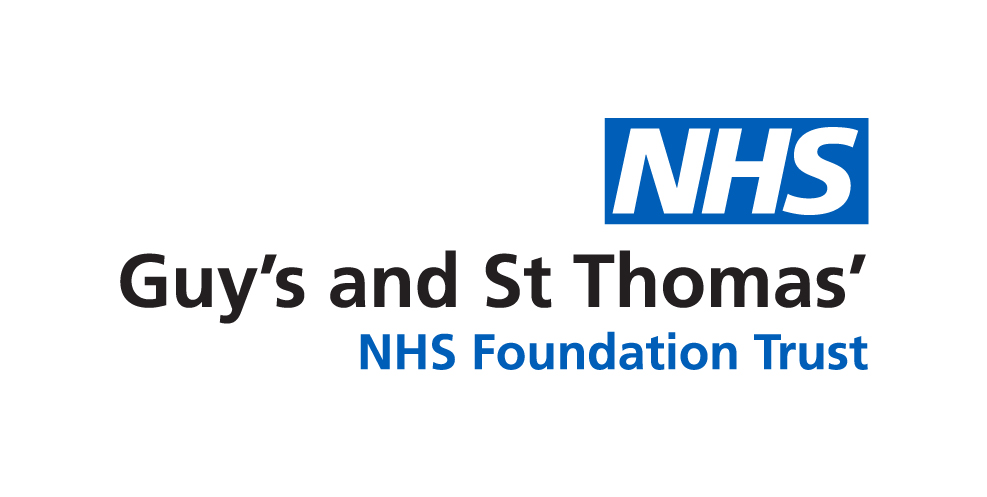
**

**King’s College London Dental Institute**

**Guy’s and St Thomas’ NHS Foundation Trust**

**Parent/Guardian Consent Form**

Version 2 (10/03/2022)

The iMAC Trial **(**Management of **i**mpacted **MA**xillary **C**entral incisors)

Name of Researcher: Professor Martyn Cobourne

IRAS Number: 280185

Please initial all boxes

1. I confirm that I have read and understood the information sheet dated xx/xx/xxxx (xx) for the above study. I have had the opportunity to consider the information, ask questions and have had these answered satisfactorily.
2. I understand that the participation of my child in this study is voluntary and that I am free to withdraw him/her at any time without giving any reason, without my child’s medical care or legal rights being affected.
3. I understand that relevant sections of my child’s medical notes and data collected during the study, may be looked at by individuals from King’s College London and Guys and St Thomas NHS Foundation Trust, regulatory authorities or from the NHS Trust. I give permission for these individuals to have access to my child’s records. As per Trust policy, these records will be stored for 25 years.
4. I agree to my child’s General Dental Practioner (GDP) being informed of my child’s participation in the study.
5. I agree to having a printed copy of the results of the study (scientific publication) posted to me following completion of the study. **(Optional)**
6. I agree that my child can take part in the above study.

Name of Participant

Name of Consenting Adult Date Signature

Name of Person Date Signature

taking consent.
