# Supplementary material for: Study protocol for the management of impacted maxillary central incisors: a multicentre randomised clinical trial: the iMAC Trial
Source: Trials. 2022 Sep 16;23:787. doi: 10.1186/s13063-022-06711-0 (PMC9479226; doi:10.1186/s13063-022-06711-0)
Supplement: Supplementary file 5 — Additional file 5: Appendix 5. Child/Young person Assent Form– The iMAC Trial. [file 13063_2022_6711_MOESM5_ESM.docx]

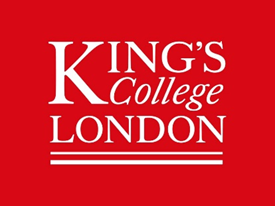
 **
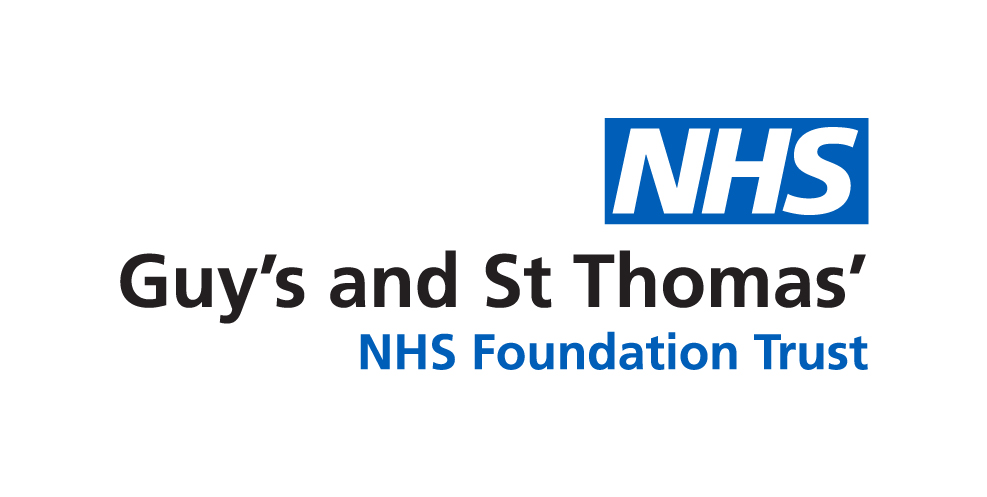
**

**King’s College London Dental Institute**

**Guy’s and St Thomas’ NHS Foundation Trust**

**Child/Young Person Assent Form (age 8-10.5 years)**

Version 2 (10/03/2022)

The iMAC Trial **(**Management of **i**mpacted **MA**xillary **C**entral incisors)

Name of Researcher: Professor Martyn Cobourne


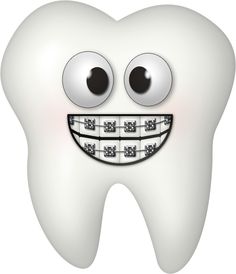


IRAS Number: 280185

**Initial boxes to agree**

| \| 1.I have read and understood the information sheet dated XX/XX/XXXX (Version 2) for the above study. The details of this study have been explained to me, I have had all my questions answered and I want to take part. \| \| --- \| |  |
| --- | --- | --- |
| \| 2. I know that it is up to me if I want to take part in this study and I can stop at any time if I want too without giving any reason, without my care or rights being affected. \| \| --- \| |  |
| \| 3. I know that my medical notes and information may be looked at by the researchers where it is important to the study. I give permission for these individuals to have access and to look my records. \| \| --- \| |  |
| \| 4. I agree to my Dentist (General Dental Practitioner) being informed that I am taking part in this study \| \| --- \| |  |
| \| 5. I understand the study and want to take part \| \| --- \| |  |

Name of Young Person (BLOCK CAPITALS) Date (dd/mmm/yyyy) Signature

Name of Person taking consent (BLOCK CAPITALS) Date (dd/mmm/yyyy) Signature

Please note - Original to be retained by the participant, 1 to be filed in the investigator file and 1 to be filed/or scanned in the patient’s medical notes.
